# Symptom clusters experienced by breast cancer patients at various treatment stages: A systematic review

**DOI:** 10.1002/cam4.3794

**Published:** 2021-03-21

**Authors:** Winnie K. W. So, Bernard M. H. Law, Marques S. N. Ng, Xiaole He, Dorothy N. S. Chan, Carmen W. H. Chan, Alexandra L. McCarthy

**Affiliations:** ^1^ The Nethersole School of Nursing Faculty of Medicine The Chinese University of Hong Kong Hong Kong SAR China; ^2^ School of Nursing, Midwifery and Social Work University of Queensland and Mater Health Services Brisbane Queensland Australia

**Keywords:** breast cancer, cancer treatment, symptom clusters, symptoms

## Abstract

Breast cancer patients often experience symptoms that adversely affect their quality of life. It is understood that many of these symptoms tend to cluster together: while they might have different manifestations and occur during different phases of the disease trajectory, the symptoms often have a common aetiology that is a potential target for intervention. Understanding the symptom clusters associated with breast cancer might usefully inform the development of effective care plans for affected patients. The aim of this paper is to provide an updated systematic review of the known symptom clusters among breast cancer patients during and/or after cancer treatment. A search was conducted using five databases for studies reporting symptom clusters among breast cancer patients. The search yielded 32 studies for inclusion. The findings suggest that fatigue‐sleep disturbance and psychological symptom cluster (including anxiety, depression, nervousness, irritability, sadness, worry) are the most commonly‐reported symptom clusters among breast cancer patients. Further, the composition of symptom clusters tends to change across various stages of cancer treatment. While this review identified some commonalities, the different methodologies used to identify symptom clusters resulted in inconsistencies in symptom cluster identification. It would be useful if future studies could separately examine the symptom clusters that occur in breast cancer patients undergoing a particular treatment type, and use standardised instruments across studies to assess symptoms. The review concludes that further studies could usefully determine the biological pathways associated with various symptom clusters, which would inform the development of effective and efficient symptom management strategies.

## 1 INTRODUCTION

Breast cancer is one of the most prevalent cancers worldwide, and patients often experience unpleasant symptoms during their treatment which adversely affect their quality of life (QOL).^1^ Previous research on the symptoms experienced by cancer patients has revealed that cancer‐associated symptoms often do not occur in isolation, and they can have a common or related aetiology, meaning that one symptom can affect the occurrence and severity of other, often related, symptoms. Therefore, research has been directed towards the exploration of groups of related cancer‐associated symptoms that occur concurrently among patients during treatment. The exploration of these symptom groups, formally defined as ‘symptom clusters’ by Kim et al.,^2^ provides useful clues for the development of strategies for symptom management, whereby symptoms may be managed simultaneously with a single intervention. This strategy could help save resources and reduce healthcare providers’ costs in caring for cancer patients. Better understanding of symptom clusters among cancer patients could also enhance the quality of care provided to affected individuals, enabling greater QOL.

Despite the increasing number of studies exploring and identifying symptom clusters experienced by breast cancer patients both during and after treatment, few published systematic reviews have summarised the findings to inform practice. Although Dong et al.^3^ conducted a systematic review on symptom clusters identified in patients with various cancer types, this review only included studies in which the participants were patients with advanced cancer. Studies identifying symptom clusters among early stage and non‐metastatic breast cancer patients were not included. Nguyen et al.^4^ also conducted a literature review on symptom clusters among breast cancer patients. However, the authors did not examine the longitudinal changes in symptom clusters patients report at various stages of the treatment trajectory. It is known, however, that symptom occurrence and severity can change during this trajectory.^5^ A summary of how symptom clusters could evolve over the course of treatment among breast cancer patients is thus required to provide insights into how symptom management strategies for cancer patients could best be tailored to each treatment stage.

The objective of this review is to provide an updated overview of the identified symptom clusters experienced by breast cancer patients during and/or after cancer treatment. The review is guided by two questions. In patients treated for breast cancer: (1) What symptom clusters occur before, during and after cancer treatment; and (2) Do the compositions of the symptom clusters, defined as the numbers and types of symptoms within the symptom clusters, change during cancer treatment?

## 2 METHODS

### 2.1 Search strategy

A literature search was conducted in May 2020. Five databases were used in the search, namely OVID MEDLINE, PubMed, EMBASE, PsycINFO and CINAHL, to identify published studies that met the eligibility criteria of the review, as set out below. A manual search using Google Scholar was also conducted to identify further eligible studies. The search strategy used for this review was as follows: ‘breast cancer’ OR ‘breast carcinoma’ OR ‘breast tumour’ OR ‘breast malignancy’ AND ‘symptom cluster’ OR ‘symptom clusters’ OR ‘multiple symptoms’ OR ‘symptom constellations’ OR ‘concurrent symptoms’ OR ‘co‐occurring symptoms’.

### 2.2 Eligibility criteria

Studies eligible for inclusion in the review were original studies of any study design that reported the identification of one or more symptom clusters within a single group of breast cancer patients at any stage in their cancer treatment trajectory. Any articles that were not original articles, or those that did not identify breast cancer‐associated symptom clusters, were excluded. Articles that were not published in English were also excluded. Moreover, as the concept of symptom clusters in oncology was first introduced in 2001,^6^ we limited the inclusion of articles to those published in or after January 2001.

### 2.3 Data extraction

After the literature search, the titles and abstracts of the identified articles were first independently screened by two authors according to the eligibility criteria. The full text of articles deemed eligible on screening was then examined to fully verify inclusion in this review. Any disagreements on eligibility were resolved by discussion between the two authors.

Data extraction was then independently conducted by two authors from the eligible studies. The extracted data comprised study settings, study design, sample size, the methodologies used in symptom cluster identification, the symptom clusters identified, the symptoms in each cluster and the instruments used for symptom assessment in the studies.

To assess the stability of symptom clusters over time, data were collected on the symptoms in the identified symptom clusters at various time points during the longitudinal studies. Differences in the compositions of these symptom clusters across time were identified by comparing the numbers and types of symptoms involved in these clusters at various time points. The presence of less than 75% of the symptoms in a particular symptom cluster at each time point of symptom assessment suggest the instability of the symptom cluster over time.^7^ Furthermore, a symptom cluster had to be present at all time points of the assessment for it to be considered stable.

As the outcomes of the included studies on symptom cluster identification generally did not contain quantitative data, and the characteristics of the participants involved in the included studies, such as the treatment received, were heterogeneous, a meta‐analysis was not performed. The review findings are presented narratively in a tabular manner.

### 2.4 Reporting quality assessment of the included studies

The quality of study reporting in the included studies was appraised using the 14‐item Standard Quality Assessment Criteria for Evaluating Primary Research Papers from a Variety of Fields developed by Kmet et al.^8^ This quality assessment tool has previously been used for critical appraisal of studies in systematic reviews of observational studies^9^ and randomised controlled trials.^10^ The items used for assessing the quality of the studies are listed in Table 1. Some of the items from the checklist were not applicable to assessing studies focused on symptom‐cluster identification, as such studies utilise methodologies of a descriptive or exploratory nature.^11^ In the assessment, studies were awarded two points for each item that was fully achieved, and one point for partial achievement of an item. Zero points were given for each item that the assessed studies failed to achieve. The total score was then calculated by summing the points awarded for each of the applicable items, and the percentage score was presented. The quality of the assessed studies was then categorised as limited (<50%), adequate (50–70%), good (70–80%) and strong (>80%), as indicated by Lee et al.^12^ Studies of limited quality were excluded from the review.

**TABLE 1:** Items included in the critical appraisal of the included studies

Item	Description of item	Item utilised in critical appraisal?
1	Research questions or objectives are sufficiently described	Yes
2	Study design is evident and appropriate	Yes
3	Method of subject / comparison group selection or source of information / input variables are described and appropriate	Yes
4	Subject characteristics are sufficiently described	Yes
5	Procedures of random allocation are described	Partially^a^
6	Procedures of blinding the investigators are described	Partially^a^
7	Procedures of blinding the subjects are described	Partially^a^
8	Outcome and exposure measures are well defined and robust to measurement or misclassification bias, and means of outcome assessment are described	Yes
9	Sample size utilised in the study is appropriate	Partially^a^
10	Analytical methods employed are justified and appropriate	Yes
11	Estimates of variance are reported in the results section	Yes
12	Confounding factors are controlled for	Partially^b^
13	Results are reported in sufficient detail	Yes
14	Conclusions drawn are supported by the results	Yes

^a^ Items that are only applicable to studies with a randomized controlled trial design, excluding those involving secondary analysis of randomized controlled trials.

^b^ Items that are not applicable to studies utilizing methodologies that are of a descriptive or exploratory nature.

The reporting quality was first assessed by one reviewer, and the assessment results were then independently verified by a second reviewer. Any disagreements in the assessment results generated by the two reviewers were resolved through discussion.

## 3 RESULTS

### 3.1 Search results

**FIGURE 1:**
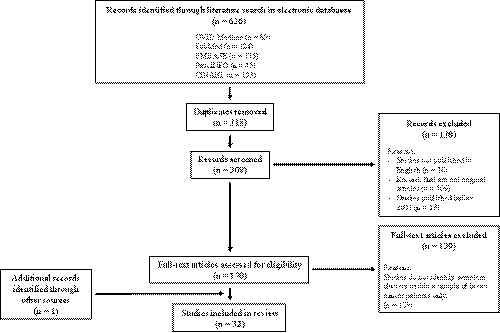
The PRISMA diagram

A total of 626 articles were initially identified through the literature search of the five databases. Moreover, through our manual search, one further original article was identified and determined to meet the eligibility criteria. Duplicated articles (*n* = 318), articles that were not original articles published in English (*n* = 125), and those that were published before January 2001 (*n* = 13) were then removed. The abstracts of the remaining 170 articles were screened to identify studies that reported the identification of symptom clusters experienced by a group of breast cancer patients. The exclusion of 139 articles reporting studies that did not fulfil this criterion left a total of 32 studies for inclusion in this review. The inclusion of these 32 studies was verified by a second author. All of the included studies attained a reporting quality score of at least 11 (a percentage score of 61%), and therefore none of the studies was excluded on the basis of low reporting quality (Table 2). Percentage agreement of the reporting quality assessment ratings was 91%, where disagreements in ratings between the two authors involved in the conduction of critical appraisal were resolved through discussion. Figure 1 provides the Preferred Reporting Items for Systematic Reviews and Meta‐Analyses (PRISMA) flow diagram that presents the results of the literature search.

**TABLE 2:** The results of the quality assessment of the included studies

Author/year	Item 1	Item 2	Item 3	Item 4	Item 5	Item 6	Item 7	Item 8	Item 9	Item 10	Item 11	Item 12	Item 13	Item 14	Quality score (% score)
Albusoul et al. (2017)	2	2	1	2	NA	NA	NA	2	NA	2	2	NA	2	2	17 (94%)
Alkathiri and Albothi (2015)	2	2	1	2	NA	NA	NA	1	NA	1	2	NA	1	1	13 (72%)
Bender et al. (2005)	2	1	0	2	NA	NA	NA	2	NA	2	0	NA	1	1	11 (61%)
Berger et al. (2018)	2	2	1	2	NA	NA	NA	2	NA	2	2	NA	2	2	17 (94%)
Bower et al. (2011)	2	0	1	2	NA	NA	NA	2	NA	2	2	2	2	2	17 (85%)
Browall et al. (2017)	2	0	1	2	NA	NA	NA	2	NA	2	0	NA	1	1	11 (61%)
Chongkham‐ang et al. (2018)	2	2	1	2	NA	NA	NA	2	NA	2	2	NA	2	2	17 (94%)
Chow et al. (2019)	2	1	0	2	NA	NA	NA	2	NA	2	2	NA	2	0	13 (72%)
Evangelista and Santos (2012)	2	1	2	2	NA	NA	NA	2	NA	2	0	NA	2	2	15 (83%)
Fu et al. (2009)	2	0	2	2	NA	NA	NA	1	NA	2	2	NA	2	2	15 (83%)
Glaus et al. (2006)	2	2	1	2	NA	NA	NA	2	NA	2	2	2	2	2	19 (95%)
Hsu et al. (2017)	2	2	2	2	NA	NA	NA	2	NA	2	2	0	2	1	17 (85%)
Kenne Sarenmalm et al. (2014)	2	2	2	2	NA	NA	NA	2	NA	2	2	NA	2	2	18 (100%)
Khan et al. (2018)	1	1	1	2	NA	NA	NA	1	NA	2	1	NA	1	2	12 (67%)
Kim et al. (2008)	2	2	2	2	NA	NA	NA	2	NA	2	2	NA	2	2	18 (100%)
Lengacher et al. (2012)	2	2	1	2	1	0	0	2	1	2	2	1	2	2	20 (71%)
Li et al. (2019)	2	2	1	2	NA	NA	NA	2	NA	2	2	NA	2	2	17 (94%)
Li et al. (2020)	2	2	1	2	NA	NA	NA	2	NA	2	2	NA	2	2	17 (94%)
Marshall et al. (2016)	2	2	1	2	NA	NA	NA	1	NA	2	0	NA	2	2	14 (78%)
Matthews et al. (2012)	2	2	1	2	NA	NA	NA	1	NA	2	2	NA	2	2	16 (89%)
Mazor et al. (2018)	2	2	1	2	NA	NA	NA	2	NA	2	2	NA	2	2	17 (94%)
Nho et al. (2018)	2	2	1	2	NA	NA	NA	2	NA	2	2	NA	2	2	17 (94%)
Phligbua et al. (2013)	2	2	1	2	NA	NA	NA	2	NA	2	2	NA	2	2	17 (94%)
Reich et al. (2017)	2	2	1	2	1	0	0	2	2	2	2	2	2	2	22 (79%)
Roiland and Heidrich (2011)	2	2	1	2	NA	NA	NA	2	NA	2	2	NA	2	2	17 (94%)
Savard et al. (2011)	2	2	1	2	NA	NA	NA	2	NA	2	2	0	2	2	17 (85%)
Starkweather et al. (2017)	1	2	1	2	NA	NA	NA	2	NA	2	2	NA	1	1	14 (78%)
Suwisith et al. (2008)	2	2	2	2	NA	NA	NA	2	NA	1	2	NA	2	2	17 (94%)
Uysal et al. (2018)	2	1	1	2	NA	NA	NA	2	NA	2	2	NA	1	0	13 (72%)
Ward Sullivan et al. (2017)	2	2	1	2	NA	NA	NA	2	NA	2	2	NA	2	2	17 (94%)
Ward Sullivan et al. (2018)	2	2	1	2	NA	NA	NA	2	NA	2	2	NA	2	2	17 (94%)
Wiggenraad et al. (2020)	2	2	1	2	NA	NA	NA	2	NA	2	2	NA	2	2	17 (94%)

### 3.2 Study characteristics

The characteristics of the 32 included studies are presented in Table 3. Inter‐rater disagreements in the extracted data occurred on 12 items shown in the table during data extraction, and these were resolved through discussion. The included studies were published between 2005 and 2020. Of these 32 studies, 13 were cross‐sectional,^13^, ^14^, ^15^, ^16^, ^17^, ^18^, ^19^, ^20^, ^21^, ^22^, ^23^, ^24^, ^25^ 11 were longitudinal,^26^, ^27^, ^28^, ^29^, ^30^, ^31^, ^32^, ^33^, ^34^, ^35^, ^36^ while the remaining eight involved a randomised clinical trial design.^37^, ^38^, ^39^, ^40^, ^41^, ^42^, ^43^, ^44^ Among these included studies, 16 involved the secondary analysis of the data of existing studies,^14^, ^42^, ^43^, ^44^ of which six were observational studies involving secondary analysis of data from randomised clinical trials.^37^, ^38^, ^39^, ^42^, ^43^, ^44^ Eleven of the included studies (34%) presented longitudinal changes in the composition of symptom clusters experienced by patients before, during and/or after cancer treatment.^27^, ^44^ One study involved a pooled, secondary data analysis of three previous studies involving participants at various stages of cancer treatment.^20^

**TABLE 3:** The characteristics of the included studies

Author/year/country	Study design	Patient characteristics/sample size	Methodology of symptom cluster identification	Instruments used for symptom assessment
Albusoul et al. (2017); USA	Secondary data analysis of a randomised controlled trial	Stage I to IIIA breast cancer patients receiving adjuvant chemotherapy (*N* = 178‐202)	Exploratory factor analysis	Hospital Anxiety and Depression Scale Symptom Experience Scale
Alkathiri and Albothi (2015); Saudi Arabia	Cross‐sectional study	Stage I to IIIA breast cancer patients receiving chemotherapy (*N* = 100)	Not specified	Symptom Experience Scale
Bender et al. (2005); USA (Study 1)^a^	Secondary data analysis of a cross‐sectional study	Stage 0 to II breast cancer patients who completed surgery and before starting adjuvant chemotherapy (*N* = 40)	Hierarchical cluster analysis	Profile of Mood States Symptom Checklist The Kupperman Index The Daily Symptom Diary
Bender et al. (2005); USA (Study 2)^a^		Stage I to III breast cancer patients who completed adjuvant chemotherapy (*N* = 88)		
Bender et al. (2005); USA (Study 3)^a^		Stage IV (metastatic) breast cancer patients with mild anaemia (*N* = 26) Patients were either receiving palliative chemotherapy or had completed chemotherapy treatment in the past		
Berger et al. (2020); USA	Secondary data analysis of a randomised controlled trial	breast cancer patients receiving surgery and chemotherapy, cancer stages not specified (*N* = 202)	Exploratory factor analysis	Hospital anxiety and depression scale Symptom experience scale
Bower et al. (2011); USA	Secondary data analysis of a cross‐sectional study	Stage 0 to IIIA breast cancer patients receiving chemotherapy and/or radiotherapy (*N* = 103)	Not specified	Fatigue symptom inventory Beck depression inventory‐II Pittsburgh Sleep Quality Index
Browall et al. (2017); Sweden	Secondary data analysis of a randomised controlled trial	Stage I to IIIA breast cancer patients receiving chemotherapy (*N* = 124)	Principal component analysis	Memorial Symptom Assessment Scale
Chongkham‐ang, et al. (2018); Thailand	Cross‐sectional study	Stage I to III breast cancer patients receiving chemotherapy (N = 322)	Exploratory factor analysis with Principal component analysis	Thai Memorial Symptom Assessment Scale
Chow et al. (2019); Canada	Longitudinal study	Stage 0 to IV breast cancer patients receiving radiotherapy (*N* = 1224)	Principal component analysis, Exploratory factor analysis and Hierarchical cluster analysis	Edmonton Symptom Assessment Scale
Evangelista and Santos (2012); Brazil	Cross‐sectional study	Stage 0 to IV breast cancer patients completed adjuvant chemotherapy and/or receiving hormone therapy (*N* = 138)	Principal component analysis	Profile of Mood States EORTC‐QLQ‐C30 EORTC‐BR23
Fu et al. (2009); USA	Cross‐sectional study	Stage 0 to III breast cancer patients completed chemotherapy, radiotherapy or hormonal therapy (*N* = 139)	Exploratory factor analysis	Memorial Symptoms Assessment Scale short form
Glaus et al. (2006); Switzerland	Cross‐sectional study	Breast cancer patients receiving hormonal therapy (cancer stage not specified) (*N* = 373)	Hierarchical cluster analysis	Clinical checklist for patients with endocrine therapy IBCSG/Linear Analogue Scales (LASA) addressing side effects of hormonal treatment and coping with disease and treatment
Hsu et al. (2017); Taiwan	Longitudinal study	Stage 0 to III breast cancer patients receiving chemotherapy (*N* = 103)	Latent class growth analysis	M. D. Anderson Symptom Inventory (Taiwan version)
Kenne Sarenmalm et al. (2014); Sweden	Secondary data analysis of a longitudinal study	Breast cancer patients receiving adjuvant chemotherapy or radiotherapy or palliative treatment (cancer stage not specified) (*N* = 206)	Principal component analysis	Memorial Symptom Assessment Scale
Khan et al. (2018); Bangladesh	Cross‐sectional study	Breast cancer patients, cancer stage and treatment received were not specified (*N* = 112)	Hierarchical cluster analysis	Symptoms identified through examinations at hospitals and documented in case sheets
Kim et al. (2008); USA	Secondary data analysis of a randomised controlled trial	Stage 0 to IV breast cancer patients receiving chemotherapy and/or radiotherapy (*N* = 282)	Common factor analysis	General Fatigue Scale Profile of mood states Pittsburgh Sleep Quality Index Side effect checklist
Lengacher et al. (2012); USA	Randomised controlled trial	Stage 0 to III breast cancer patients completed chemotherapy and/or radiotherapy (*N* = 82)	Hierarchical cluster analysis	M.D. Anderson Symptom Inventory
Li et al. (2019); USA	Secondary data analysis of a longitudinal study	Stage I to IIIA breast cancer patients receiving surgery with or without chemotherapy (*N* = 339)	Exploratory factor analysis	Breast Cancer Prevention Trial Symptom Checklist Profile of mood states Brief pain inventory‐short form Beck Depression Inventory‐II Patient's assessment of own functioning
Li et al. (2020); USA	Secondary data analysis of a longitudinal study	Stage I to IIIA breast cancer patients receiving surgery with or without chemotherapy (*N* = 354)	Exploratory factor analysis	Breast cancer prevention trial symptom checklist Profile of mood states Beck depression inventory‐II Patient's assessment of own functioning
Marshall et al. (2016); USA	Secondary data analysis of a cross‐sectional study	Data from MedHelp.org breast cancer forum: Breast cancer patients completed cancer treatment (treatment not specified) (*N* = 12,991) Data from research study: Stage I to III breast cancer patients completed chemotherapy or radiotherapy (*N* = 653)	K‐medoid clustering	Symptom checklist derived from the Women's Health Initiative (used in symptom assessment in the research study only)
Matthews et al. (2012); USA	Secondary data analysis of a cross‐sectional study	Stage I to IV breast cancer patients receiving radiotherapy (*N* = 93)	Confirmatory factor analysis	Symptom Distress Scale
Mazor et al. (2018); USA	Secondary data analysis of a longitudinal study	Stage 0 to IV breast cancer patients receiving surgery (*N* = 398)	Exploratory factor analysis	Self‐administered comorbidity questionnaire Menopausal Symptoms Scale
Nho et al. (2018); South Korea	Cross‐sectional study	Stage 0 to IV breast cancer patients completed surgery, chemotherapy, radiotherapy and/or hormone therapy (*N* = 241)	Principal component analysis	EORTC QLQ‐C30 EORTC QLQ‐BR23 Hospital Anxiety and Depression Scale
Phligbua et al. (2013); Thailand	Longitudinal study	Stage I to IIIA breast cancer patients receiving chemotherapy (*N* = 112)	Exploratory factor analysis	The Modified Memorial Symptom Assessment Scale
Reich et al. (2017); USA	Randomised controlled trial	Stage 0 to III breast cancer patients completed chemotherapy and/or radiotherapy (*N* = 299)	Exploratory factor analysis	The Center for Epidemiological Studies Depression Scale State‐trait anxiety inventory Perceived Stress Scale M.D. Anderson Symptom Inventory Pittsburgh Sleep Quality Index Fatigue Symptom Inventory Brief pain inventory
Roiland and Heidrich (2011); USA	Secondary data analysis of a randomised controlled trial	Breast cancer patients completed chemotherapy, radiotherapy or hormonal therapy (cancer stage not specified) (*N* = 192)	Exploratory factor analysis and confirmatory factor analysis	Symptom Bother Scale–Revised
Savard et al. (2011); Canada	Longitudinal study	Stage I to III breast cancer patients receiving chemotherapy and/or radiotherapy (*N* = 58)	Canonical correlation analysis	Insomnia Severity Index Hot flush diary
Starkweather et al. (2017); USA	Longitudinal study	Stage I to IIIA breast cancer patients receiving adjuvant chemotherapy (*N* = 75)	Exploratory factor analysis	Hospital Anxiety and Depression Scale Brief fatigue inventory General Sleep Disturbance Scale Brief pain inventory Perceived Stress Scale CNS vital signs™ (software for assessing cognition)
Suwisith et al. (2010); Thailand	Cross‐sectional study	Stage I to IV breast cancer patients receiving chemotherapy (*N* = 320)	Not specified	Memorial Symptoms Assessment Scale
Uysal et al. (2019); Turkey	Cross‐sectional study	Stage I to IV breast cancer patients completed surgery and/or receiving chemotherapy (*N* = 170)	Hierarchical clustering analysis	Memorial Symptom Assessment Scale
Ward Sullivan et al. (2017); USA	Secondary data analysis of a longitudinal study	Breast cancer patients receiving adjuvant chemotherapy (cancer stage not specified) (*N* = 515)	Exploratory factor analysis	Memorial Symptom Assessment Scale
Ward Sullivan et al. (2018); USA	Secondary data analysis of a longitudinal study	Breast cancer patients receiving chemotherapy, cancer stage not specified (*N* = 540)	Exploratory factor analysis	Memorial Symptom Assessment Scale
Wiggenraad et al. (2020); Sweden	Secondary data analysis of a randomised controlled trial	Stage I to IIIA breast cancer patients receiving chemotherapy (*N* = 206)	Principal component analysis	Memorial Symptom Assessment Scale

^a^ Bender et al. (2005) study consists of three independent studies using three different samples of participants.

The sample size of the included studies ranged from 26 to 12,991, with the latter number being the sample size used in a study that involved a secondary analysis of data obtained from users of an online health forum.^17^

### 3.3 The commonly identified symptom clusters of breast cancer patients at different stages of cancer treatment

The composition of the symptom clusters identified in the included studies of breast cancer patients before, during and after cancer treatment (either curative treatments, adjuvant therapies or surgery) are presented in Tables 4, 5 and 6, respectively. As the naming of symptom clusters varied across studies, a broad title that described the nature of the core symptoms in each cluster was given to facilitate interpretation. The specific names of the symptom clusters that were reported in the included studies are highlighted with quotation marks.

**TABLE 4:** Some commonly identified symptom clusters and other symptom clusters among breast cancer patients before they underwent cancer treatment.

Author/year	Notes on how symptoms were assessed	Pain‐Fatigue‐Sleep disturbance	The Menopausal Cluster (hot flashes‐sweats/night sweats)	The Psychological Cluster (sadness‐worry‐anxiety‐depression)	Other clusters identified
Albusoul et al. (2017)	N/A	**Yes** + Nausea, appetite, bowel pattern ‐ Sleep disturbance			Sleep disturbance, concentration, anxiety, appearance
Bender et al. (2005) (study 1)	N/A			**Yes** + Nervousness ‐ Sadness, worry	Fatigue, lack of energy, decreased physical strength (weakness) Memory problems, loss of concentration Difficulty sleeping, aching muscles and joints, backaches
Berger et al. (2020)	N/A	**Yes** + Nausea, bowel pattern ‐ Sleep disturbance			Sleep disturbance, concentration, anxiety
Browall et al. (2017)	N/A			**Yes** + Difficulty concentrating ‐ Anxiety, depression	Taste change, constipation, diarrhoea Breathlessness, dizziness, dry mouth, nausea
Chow et al. (2019)	PCA			**Yes** + Well‐being ‐ Sadness, worry	Pain, tiredness, nausea, drowsiness, loss of appetite, dyspnoea
	EFA			**Yes** + Well‐being ‐ Sadness, worry	Tiredness, drowsiness, pain, nausea, loss of appetite, dyspnoea
	HCA			**Yes** + Well‐being ‐ Sadness, worry	Pain, tiredness, drowsiness, dyspnoea Nausea, loss of appetite
Kim et al. (2008)	N/A	**Yes** + Depression, cognitive disturbance			
Li et al. (2020)			**Yes**	**Yes** + Changes in sleep patterns, avoid of social affairs, fatigue ‐ Sadness, worry	Difficulty concentrating, easily distracted, forgetfulness, perceived cognitive disturbance Joint pain, general aches and pain, muscle stiffness Difficulty with bladder control when laughing or crying, difficulty with bladder control at other times Vaginal dryness, pain with intercourse Decreased appetite, weight loss
Mazor et al. (2018)	Based on symptom occurrence		**Yes** + Vaginal dryness	**Yes** + Difficulty concentrating, difficulty falling asleep, fatigue, wake during the night, waking too early ‐ Sadness, worry	Anger, impatience, irritability, mood swings, tension Backache/neckache, general body aches, joint pain or stiffness, numbness or tingling, painful/tender breast, weight gain
	Based on symptom severity		**Yes** + General body aches, vaginal dryness, numbness/tingling, weight gain	**Yes** + Anger, difficulty concentrating, difficulty falling asleep, fatigue, forgetfulness, headache, impatience, irritability, mood swings, tension, waking during the night, waking too early ‐ Sadness, worry	General body aches, numbness/tingling, backache/neckache, joint pain and stiffness
Phligbua et al. (2013)	N/A		**Yes** + Mood swings, feeling irritable, difficulty concentrating	**Yes** ‐ Anxiety, depression	Dizziness, joint pain, vaginal itching/irritation, constipation Cough, itchiness, numbness/tingling in hands and feet Difficulty sleeping, lack of energy
Starkweather et al. (2017)	N/A	**Yes** + Verbal memory		**Yes** + Perceived stress, sleep disturbance, fatigue ‐ sadness, worry	Cognitive flexibility, executive functioning, complex attention, reaction time, processing speed Psychomotor speed, visual memory, processing speed
Ward Sullivan et al. (2018)	Based on symptom occurrence		**Yes**	**Yes** + Difficulty concentrating, nervousness, irritability, ‘I don't look like myself’ ‐ Anxiety, depression	Pain, dry mouth, nausea, drowsiness, numbness/tingling, lack of appetite, dizziness Difficulty sleeping, abdominal cramps, shortness of breath, weight loss Weight loss, weight gain (weight changes) Weight gain, mouth sores, hair loss, change in the way food tastes, change in skin
	Based on symptom severity		**Yes**	**Yes** + Difficulty concentrating, nervousness, irritability, ‘I don't look like myself’ ‐ Anxiety, depression	Pain, dry mouth, nausea, drowsiness, dizziness Feeling bloated, diarrhoea, abdominal cramps Lack of appetite, weight gain, weight loss (weight changes) “I don't look like myself”, weight gain, hair loss, change in the way food tastes, changes in skin

Abbreviations: EFA, exploratory factor analysis; HCA, hierarchical cluster analysis; PCA, principal component analysis.

**TABLE 5:** Some commonly identified symptom clusters and other symptom clusters among breast cancer patients undergoing cancer treatment.

Author/year	Notes on when/how symptoms were assessed	The gastrointestinal cluster (nausea‐lack of appetite)	Pain‐fatigue‐sleep disturbance	The psychological cluster (anxiety‐depression‐worry‐sadness‐nervousness‐feeling irritable)	Other clusters identified
Albusoul et al. (2017)	At cycle 3 of chemotherapy	**Yes**	**Yes** + Bowel pattern, loss of concentration, appearance, anxiety, depression	**Yes** + Bowel pattern, loss of concentration, appearance, pain, fatigue, sleep disturbance ‐ Worry, sadness, nervousness, feeling irritable	
	At cycle 4 of chemotherapy		**Yes** + Bowel pattern, nausea ‐ Fatigue	**Yes** + Fatigue, lack of appetite, loss of concentration, appearance ‐ Worry, sadness, nervousness, feeling irritable	
Alkathiri and Albothi (2015)	Cluster identification based on symptom severity	**Yes**	**Yes** + Concentration, bowel pattern, appearance		
	Cluster identification based on symptom frequency and distress	**Yes** + Sleep disturbance	**Yes** + Concentration, bowel pattern, appearance ‐ Sleep disturbance		
Bender et al. (2005) (study 3)	Not specified			**Yes** + Fatigue, decreased physical strength (weakness), lack of energy, loss of concentration ‐ Worry, sadness, feeling irritable	
Browall et al. (2017)	After cycle 1 of chemotherapy			**Yes** + Difficulty concentrating ‐ Nervousness, feeling irritable, anxiety, depression	Lack of appetite, taste change, constipation, diarrhoea Breathlessness, dizziness, dry mouth, nausea, hair loss
	After cycle 3 of chemotherapy			**Yes** ‐ Nervousness, feeling irritable, anxiety, depression	Mouth sore, dry mouth Lack of appetite, breathlessness, nervousness, lack of energy, feeling irritable, dizziness
Chongkham‐ang et al. (2018)	Cluster identification based on symptom severity	**Yes** + Vomiting, difficulties swallowing, feeling bloated, dizziness, lack of energy, shortness of breath		**Yes** + Sleep difficulties, difficulties concentrating, drowsiness, sweats ‐ Anxiety, depression	Change in skin, hair loss, I don't look like myself, mouth sores, change in the way food tastes, weight loss, constipation, dry mouth Pain, numbness/tingling in hands and feet, itching, problems in urination, cough
	Cluster identification based on symptom distress	**Yes** + Vomiting, difficulty swallowing, dizziness		**Yes** + Sleep difficulties, difficulty concentrating, lack of energy, drowsiness, pain, numbness/tingling in hands and feet, shortness of breath, sweats ‐ Anxiety, depression	I don't look like myself, changes in skin, hair loss Itching, mouth sores, constipation, dry mouth, problems with urination, weight loss, cough, feeling bloated, change in the way food tastes
Glaus et al. (2006)	Not specified				Hot flashes, weight‐gain, tiredness/fatigue, reduced sexual interest, vaginal dryness
Hsu et al. (2017)	After cycle 3 of chemotherapy				Pain, shortness of breath, vomiting, memory problems, numbness or tingling Nausea, disturbed sleep, distress/upset, drowsiness, sadness Fatigue, lack of appetite, dry mouth
Kenne Sarenmalm et al. (2014)	At baseline when participants were receiving chemotherapy			**Yes** + Sleep difficulties, reduced quality of life, reduced health status ‐ Feeling irritable, anxiety, depression	Drowsiness, dry mouth, lack of appetite, feeling irritable, difficulty swallowing, shortness of breath Weight loss, taste change, constipation, vomiting, hair loss, nausea
	At 1‐month follow‐up	**Yes** + Taste changes, vomiting, constipation, weight loss		**Yes** + Difficulty concentrating, ‘I don't look like myself, lack of energy, reduced quality of life, sleep difficulties ‐ Anxiety, depression	Changes in skin, swelling in arms/legs, feeling bloated, numbness/tingling and hands and feet, itching
	At 3‐month follow‐up	**Yes** + Taste changes, vomiting, hair loss, weight loss		**Yes** + Difficulty concentrating, reduced quality of life, lack of energy, ‘I don't look like myself, reduced health status ‐ Anxiety, depression	Changes in skin, itching, pain, difficulty swallowing
	At 6‐month follow‐up			**Yes** + Sweats, pain, problems with sexual interest, feeling bloated, difficulty sleeping, reduced quality of life ‐ Anxiety, depression	Taste changes, drowsiness, lack of appetite, lack of energy, dry mouth, hair loss, difficulty concentrating Changes in skin, vomiting, mouth sores, swelling of arms and legs, difficulty swallowing
Kim et al. (2008)	After 2^nd^ cycle of chemotherapy or at the final week of the radiotherapy course	**Yes** + Vomiting	**Yes** + Depression, cognitive disturbance, hot flashes		
	After 3^rd^ cycle of chemotherapy	**Yes** + Vomiting	**Yes** + Depression, cognitive disturbance		
Li et al. (2020)	6 months after start of adjuvant therapy			**Yes** + Fatigue, avoid of social affairs ‐ Worry, sadness, nervousness, feeling irritable	Difficulty concentrating, forgetfulness, easily distracted, perceived cognitive disturbance, dry mouth Joint pain, general aches and pain, muscle stiffness Night sweats, hot flashes Vaginal dryness, pain with intercourse Diarrhoea, nausea Difficulty with bladder control when laughing or crying, difficulty with bladder control at other times Unhappy with the appearance of my body, weight gain
	12 months after start of adjuvant therapy				Fatigue, depression, changes in sleep patterns Easily distracted, difficulty concentrating, perceived cognitive disturbance, forgetfulness, excitability, tendency toward accidents, short temper, anxiety Joint pain, general aches and pain, muscle stiffness Night sweats, hot flashes Difficulty with bladder control when laughing or crying, difficulty with bladder control at other times Vaginal dryness, pain with intercourse Unhappy with the appearance of my body, weight gain
	18 months after start of adjuvant therapy			**Yes** + Perceived cognitive disturbance, excitability, forgetfulness, difficulty concentrating, easily distracted, fatigue ‐ Worry, sadness, nervousness, feeling irritable	Joint pain, general aches and pain, muscle stiffness Night sweats, hot flashes Difficulty with bladder control when laughing or crying, difficulty with bladder control at other times Vaginal dryness, pain with intercourse Unhappy with the appearance of my body, weight gain
Matthews et al. (2012)	At least 3 weeks after radiotherapy initiation	**Yes** + Bowel problems	**Yes**		Concentration, appearance, outlook
Phligbua et al. (2013)	After cycle 1 of chemotherapy	**Yes** + Lack of energy, drowsiness, dizziness, taste change		**Yes** + Pain ‐ Worry, sadness, anxiety, depression	'I don't look like myself’, worry, difficulty concentrating, hair loss, skin changes Constipation, urinary problem, difficulty sleeping, feeling bloated Mouth sore, dry mouth
Savard et al. (2011)	Not specified				Hot flashes, insomnia
Starkweather et al. (2017)	Before cycle 4 of chemotherapy		**Yes** + Perceived stress, anxiety, depression ‐ Pain	**Yes** + Perceived stress, fatigue, sleep disturbance ‐ Worry, sadness, nervousness, feeling irritable	Cognitive flexibility, executive functioning, complex attention, reaction time, processing speed, psychomotor speed, pain Processing speed, psychomotor speed, pain, verbal memory Psychomotor speed, visual memory
Suwisith et al. (2010)	Cluster identification based on symptom severity			**Yes** + ‘I don't look like myself’, difficulty concentrating, sleep difficulties, sweats, constipation ‐ Anxiety, depression	vomiting, lack of energy, lack of appetite, dizziness, drowsiness, shortness of breath, feeling bloated hair loss, taste change, mouth sore, skin change, difficulty swallowing numbness/tingling, pain, dry mouth
	Cluster identification based on symptom distress	**Yes** + Vomiting, lack of energy, dizziness, drowsiness		**Yes** + Difficulty concentrating, sleep difficulties, numbness/tingling, shortness of breath, feeling bloated, sweats, pain ‐ Anxiety, depression	Mouth sore, hair loss, skin change, taste change, difficulty swallowing, constipation, dry mouth, ‘I don't look like myself’
Uysal et al. (2019)	Not specified			**Yes** + Sleep difficulties ‐ Anxiety, depression	Pain, lack of energy, drowsiness, sweat, swelling of arms or legs Nausea, feeling bloated, taste change, hair loss, constipation Vomiting, diarrhoea, problems with sexual activity, lack of appetite, dizziness, weight loss
Ward Sullivan et al. (2017)	1 week after initiation of chemotherapy	**Yes** + Dry mouth, taste change, weight loss, abdominal cramps, diarrhoea		**Yes** + ‘I don't look like myself’ ‐ Anxiety, depression	Hot flashes, difficulty sleeping, sweats, problems with sexual interest or activity Weight loss, weight gain (weight changes), feeling bloated ‘I don't look like myself’, taste change, hair loss, mouth sores
	Cluster identification based on symptom occurrence				
	1 week after initiation of chemotherapy	**Yes** + Weight loss		**Yes** ‐ Anxiety, depression	Hot flashes, sweats Weight gain, feeling bloated, abdominal cramp ‘I don't look like myself, taste change, hair loss, mouth sores, skin changes Drowsiness, tingling/numbness in hands/feet, pain
	Cluster identification based on symptom severity				
Ward Sullivan et al. (2018)	1 week after start of chemotherapy	**Yes** + Dry mouth, taste change, weight loss, abdominal cramps, diarrhoea		**Yes** + ‘I don't look like myself’ ‐ Anxiety, depression	Hot flashes, difficulty sleeping, sweats, problem with sexual interest or activity Weight loss, weight gain (weight changes), feeling bloated, “I don't look like myself”, taste change, hair loss, mouth sores
	Cluster identification based on symptom occurrence				
	1 week after start of chemotherapy	**Yes** + Weight loss, weight gain (weight changes)		**Yes** ‐ Anxiety, depression	hot flashes, sweats Weight gain, feeling bloated, abdominal cramp ‘I don't look like myself’, taste change, hair loss, mouth sores, skin changes Drowsiness, tingling/numbness in hands/feet, pain
	Cluster identification based on symptom severity				
	2 weeks after start of chemotherapy	**Yes** + Weight gain, weight loss (weight changes), taste change		**Yes** + Difficulty concentrating, drowsiness ‐ Anxiety, depression	Hot flashes, sweats Abdominal cramps, difficulty sleeping, feeling bloated, weight gain, nausea Taste change, changes in skin, itching, mouth sores, “I don't look like myself”
	Cluster identification based on symptom occurrence				
	2 weeks after start of chemotherapy	**Yes** + Weight gain, weight loss (weight changes), taste change		**Yes** + Difficulty concentrating, drowsiness ‐ Anxiety, depression	Hot flashes, sweats Feeling bloated, abdominal cramps, weight gain Taste change, mouth sores, hair loss, “I don't look like myself”, changes in skin
	Cluster identification based on symptom severity				
Wiggenraad et al. (2020)				**Yes** + Lack of appetite, pain, difficulty sleeping, shortness of breath, I don't look like myself ‐ Worry, anxiety, depression	Lack of energy, difficulty concentrating, feeling bloated, diarrhoea, worry, drowsiness, nausea Hair loss, taste change, sweats

**TABLE 6:** Some commonly identified symptom clusters and other symptom clusters among breast cancer patients after completion of cancer treatment.

Author/year	Notes on when/how symptom clusters were identified	Fatigue‐Sleep disturbance	The psychological cluster (depression‐anxiety)	The gastrointestinal cluster (Nausea‐lack of appetite‐diarrhoea)	The menopausal cluster (hot flashes‐vaginal dryness‐night sweats)	Other clusters identified
Albusoul et al. (2017)	N/A	**Yes** + Pain				Concentration, appearance, anxiety
Bender et al. (2005) (Study 2)	N/A		**Yes** + Fatigue, lack of energy, weakness, headaches, problems with memory, loss of concentration			
Berger et al. (2020)	1 month after last chemotherapy cycle	**Yes** + Concentration				Concentration, appearance, anxiety
	6 months after last chemotherapy cycle	**Yes** + Concentration, anxiety				
Bower et al. (2011)	N/A	**Yes** + Depression				
Browall et al. (2017)	N/A			**Yes** + Taste change, constipation ‐ Nausea		Nervousness, worry, sadness Problems with sexual relations, sweats, difficulty sleeping
Chow et al. (2019)	1 week post‐treatment/PCA		**Yes** + Pain, tiredness, well‐being	**Yes** + Drowsiness, dyspnoea ‐ Diarrhoea		
	1 week post‐treatment/EFA		**Yes** + Pain, well‐being	**Yes** + Tiredness, drowsiness, dyspnoea		
	1 week post‐treatment/HCA		**Yes** + Pain, well‐being	**Yes** ‐ Diarrhoea		Tiredness, drowsiness, dyspnoea
	142 days post‐treatment on average/PCA		**Yes** + Pain, tiredness, well‐being, drowsiness, dyspnoea	**Yes** ‐ Diarrhoea		
	142 days post‐treatment on average/EFA		**Yes** + Pain, tiredness, well‐being, drowsiness, dyspnoea, nausea, loss of appetite			
	142 days post‐treatment on average/HCA		**Yes**	**Yes** ‐ Diarrhoea		Pain, tiredness, drowsiness, well‐being, dyspnoea
Evangelista and Santos (2012)	N/A			**Yes** + Vomiting		Depression, confusion, anger, tension, fatigue, breast symptoms Pain, breathing difficulties, arm symptoms, insomnia
Fu et al. (2009)	N/A		**Yes** + Grief/loss	**Yes** + Lymphedema, neuropathy ‐ Diarrhoea		Fatigue, poor sex drive, hot flashes, headache, poor memory Sleep disturbance, muscle ache, bone pain
Khan et al. (2018)	N/A		**Yes** + Pain, weakness, sleeplessness, loss of appetite			Cough, breathlessness, nausea, constipation Lymphedema, sadness
Lengacher et al. (2012)	N/A	**Yes** + Drowsiness		**Yes** + Vomiting, shortness of breath, dry mouth, numbness ‐ Diarrohea		Distress, sadness, pain, remembering
Li et al. (2019)	N/A		**Yes** + Fatigue, avoidance of social affairs + Change in sleep pattern (for patients receiving surgery only)	**Yes** ‐ Lack of appetite	**Yes** ‐ Vaginal dryness	Easily distracted, perceived cognitive impairment, difficulty concentrating, forgetfulness Joint pain, muscle stiffness, general ache, general pain severity (+ hand swelling for patients not receiving chemotherapy) Pain with intercourse, vaginal dryness Reduced appetite, weight loss Difficulty with bladder control when laughing or crying, difficulty with bladder control at other times
Marshall et al. (2016)	Clusters identified through the breast cancer forum data			**Yes** + Abdominal pain, constipation ‐ Lack of appetite	**Yes** + Joint pain, weight gain, mood changes, depression	General aches, fatigue, headache, muscle pain, neck‐skull aches Sleep too much, difficulty concentrating, feeling bloated
	Clusters identified with symptoms reported to be of **moderate or severe symptom severity** in research study	**Yes** + Headache, sleep too much, mood changes, nausea, abdominal pain, constipation			**Yes**	Decreased efficiency, avoid social affairs, diarrhoea, loss of interest in work, feeling bloated, depression, lowered work performance, difficulty concentrating Increased appetite, increased weight General aches, joint pain, muscle pain, neck‐skull pain
	Clusters identified with symptoms reported to be of **severe symptom severity only** in research study				**Yes** + Restless sleep	General aches, muscle pain, neck‐skull aches, joint pain, sleep too much Fatigue, lowered work performance, depression, nausea, constipation, feeling bloated, avoid social affairs, loss of interest in work, headache, difficulty concentrating, decreased efficiency, restless sleep Abdominal pain, diarrhoea Increased appetite, increased weight
Mazor et al. (2018)	Clusters identified based on symptom occurrence		**Yes** + Anger, difficulty concentrating, fatigue, forgetfulness, impatience, irritability, mood swings, tension		**Yes** + Daytime sweats	Backache/neckache, general body aches, joint pain or stiffness Difficulty falling asleep, wake during the night, waking too early
	Clusters identified based on symptom severity		**Yes** + Anger, impatience, irritability, mood swings, tension		**Yes** + Daytime sweats	Difficulty concentrating, fatigue, forgetfulness, painful/tender breasts General body aches, headache, backache/neckache, joint pain and stiffness Difficulty falling asleep, wake during the night, wake too early
Nho et al. (2018)	N/A	**Yes** + Anxiety, depression, loss of appetite, dyspnoea	**Yes** + Fatigue, sleep disturbance, loss of appetite, dyspnoea			Arm symptoms, breast symptoms, pain, systemic therapy side effects, nausea/vomiting and constipation
Phligbua et al. (2013)	N/A				**Yes** + Sleep difficulties, sweat, difficulty concentrating, pain, worry	Lack of energy, drowsiness, lack of appetite, taste change Mood swings, feeling irritable, joint pain Numbness/tingling in hands/feet, dry mouth, vaginal dryness Skin changes, hair loss, ‘I don't look like myself’
Reich et al. (2017)	N/A	**Yes** + Drowsiness	**Yes** + Stress, emotional well‐being			Mindfulness, memory
Roiland and Heidrich (2011)	N/A		**Yes** + Mood changes, nightmares, headache, hot flashes, vaginal dryness, weight gain or loss			Aching, stiffness, pain, joint pain, weakness, fatigue Balance problem, dizziness, memory problems, difficulty concentrating Dry skin, dry mouth, itchiness, thirst, shortness of breath Incontinence (i.e. leaky bladder), increased urination, decreased sex drive, irritated eyes Swelling in hands/feet, changes in smell/taste, hair loss, constipation, lymphedema, numbness in hands/feet Wake too often, wake too early, difficulty falling asleep, vaginal discharge
Starkweather et al. (2017)	N/A	**Yes** + Pain, perceived stress, anxiety, depression	**Yes** + Perceived stress, pain, fatigue, sleep disturbance			Cognitive flexibility, executive functioning, complex attention, reaction time Processing speed, reaction time, psychomotor speed, pain, fatigue Psychomotor speed, verbal memory, visual memory

Abbreviations: EFA, exploratory factor analysis; HCA, hierarchical cluster analysis; PCA, principal component analysis.

#### 3.3.1 Identified symptom clusters among patients prior to undergoing treatment

Among the included studies, 11 (34%) had identified the symptom clusters experienced by breast cancer patients before they received primary and/or adjuvant treatments for cancer. In total, three symptom clusters were found to be commonly reported in at least four of these 11 studies. These clusters included Pain‐Fatigue‐Sleep disturbance, the Menopausal Cluster and the Psychological Cluster (Table 4).

Pain‐Fatigue‐Sleep disturbance was found in four studies examining symptom clusters among patients prior to undergoing treatment,^30^, ^37^, ^39^, ^44^ although the data obtained by Albusoul et al. and Berger et al. showed that sleep disturbance was not associated with the other two symptoms in the cluster. All four of the studies showed that additional symptoms were also associated with this symptom cluster.

Experiencing hot flushes was found to form a cluster with night sweats or sweats (the Menopausal Cluster) in four studies.^28^, ^33^, ^34^, ^35^ Whilst Li et al. and Ward Sullivan et al. revealed a clustering of hot flushes and night sweats/sweats, Mazor et al. and Phligbua et al. reported that further symptoms were associated with this symptom cluster, such as mood swings, irritability, difficulty concentrating, body aches, weight gain, numbness/tingling and vaginal dryness.

Finally, at least two of the following psychological symptoms, namely sadness, worry, anxiety and depression (the Psychological Cluster), were shown in eight studies to co‐occur in patients prior to receiving treatment.^20^, ^28^, ^30^, ^32^, ^33^, ^34^, ^35^, ^38^ Interestingly, (fatigue and/or sleep disturbance), were also shown to exhibit an association with some of the symptoms in this cluster, namely anxiety and depression,^30^, ^33^, ^34^ suggesting that the symptoms in both clusters may mutually influence their occurrence. Similar to the previous two clusters, this symptom cluster was also found to co‐occur with other symptoms, as shown in Table 4.

#### 3.3.2 Identified symptom clusters among patients who were undergoing treatment

Nineteen studies (59%) investigated the symptom clusters reported by breast cancer patients who were undergoing cancer treatment. Five of these studies reported symptom clusters at more than one time point during cancer treatment at which symptom assessment was conducted.^27^, ^33^, ^35^, ^37^, ^38^ Furthermore, five studies reported the symptom clusters on the basis of multiple symptom parameters, such as symptom distress, symptom occurrence and symptom severity.^13^, ^15^, ^21^, ^31^, ^35^ Of the 19 studies that investigated symptom clusters among those undergoing treatment, the most commonly reported clusters were the Gastrointestinal Cluster (nausea‐lack of appetite), Pain‐Fatigue‐Sleep disturbance and the Psychological Cluster (anxiety‐depression‐worry‐sadness‐nervousness‐irritability) (Table 5).

Nausea‐lack of appetite (the Gastrointestinal Cluster) in breast cancer patients receiving cancer treatment was reported in 10 studies.^13^, ^14^, ^15^, ^39^ All except two ^13^, ^37^ of these studies showed that additional symptoms were associated with this symptom cluster. Interestingly, in one study, this symptom cluster was identified only when symptom cluster identification was based on symptom distress levels, and not when it was based on symptom severity levels.^15^ Likewise, Alkathiri and Albothi,^13^ Chongkham‐ang et al.^21^ and Ward Sullivan et al.^31^, ^35^ demonstrated that the number and/or types of additional symptoms that were associated with this symptom cluster could vary as a result of the parameters used in symptom cluster identification. These observations suggest that the procedures used in symptom cluster identification could result in variations in the identified clusters.

Five studies reported the co‐occurrence of Pain‐Fatigue‐Sleep disturbance among patients during cancer treatment.^13^, ^14^, ^30^, ^37^, ^39^ This cluster was identified to exist independently by Matthews et al.,^14^ while the remaining studies reported that additional symptoms can also form clusters with pain, fatigue and sleep disturbance. Further, Alkathiri and Albothi reported that variations in the additional symptoms that contribute to this symptom cluster were the result of differences in the dimensions used for symptom clustering, namely symptom severity, symptom frequency and symptom distress.^13^

Thirteen studies^15^, ^20^, ^21^, ^25^, ^27^, ^28^, ^30^, ^31^, ^33^, ^35^, ^37^, ^38^, ^43^ demonstrated that psychological symptoms such as anxiety, depression, worry, sadness, nervousness and irritability were commonly experienced by patients undergoing cancer treatment, and some of these psychological symptoms could even co‐occur, which demonstrated the potential of these five symptoms to form a symptom cluster (the Psychological Cluster). Due to the larger number of symptoms in the Psychological Cluster, it was less consistently reported in these studies. In each of these 13 studies, at least one of the aforementioned six symptoms in this cluster was absent. Moreover, most of these studies reported that additional symptoms were associated with this cluster. Notably, Li et al.^33^ showed that this symptom cluster exhibited a certain degree of longitudinal change over the course of an adjuvant therapy involving the use of anastrozole, with or without chemotherapy. Two of the symptoms in this cluster (anxiety and depression) were found to form a cluster at both six and 18 months after the initiation of the adjuvant therapy, However, after the patients had received this therapy for 12 months, the factor loading of these two symptoms was insufficient to form a cluster. Likewise, the composition of this symptom cluster appeared to change between six and 18 months after initiating therapy, as indicated by the differences between the numbers of symptoms that exhibited associations with the two psychological symptoms at those time points. This set of observations suggested the dynamic nature of symptom cluster composition during the course of cancer treatment.

#### 3.3.3 Identified symptom clusters among patients who had completed treatment

Among the included studies, 18 (56%) examined the symptom clusters experienced by patients who had completed cancer treatment. The most commonly reported symptom clusters in these studies were fatigue‐sleep disturbance, depression‐anxiety (the Psychological Cluster), nausea‐lack of appetite‐diarrhoea (the Gastrointestinal Cluster) and hot flushes‐vaginal dryness‐night sweats (the Menopausal Cluster) (Table 6).

Eight studies examining the symptom clusters reported by patients who had completed breast cancer treatment reported the clustering of fatigue and sleep disturbance.^17^, ^18^, ^24^, ^30^, ^37^, ^40^, ^41^, ^44^ Only two of these studies reported that pain was associated with this symptom cluster.^30^, ^37^ None of these eight studies reported the independent existence of this symptom cluster. Notably, Marshall et al.^17^ reported the identification of this symptom cluster only when such identification was performed using symptoms reported to be moderate or severe by patients, and not when only symptoms that were rated severe were included.

The symptom cluster of depression‐anxiety (the Psychological Cluster) among breast cancer patients who had completed treatment was reported in 10 studies.^19^, ^20^, ^23^, ^24^, ^30^, ^32^, ^34^, ^36^, ^40^, ^42^ None of these studies showed that this cluster existed independently, except Chow et al.,^32^ who used hierarchical cluster analysis for symptom clustering. Chow et al. also found slight changes in the composition of this symptom cluster, in terms of the additional symptoms that clustered with depression‐anxiety when different methodologies of cluster analysis were used. However, Starkweather et al.^30^ and Khan et al.^23^ found that depression‐anxiety could cluster with fatigue‐sleep disturbance, together with other symptoms, suggesting that there might be an interaction or association between these two symptom clusters. Furthermore, Roiland and Heidrich^42^ found that depression‐anxiety could co‐occur and be associated with certain menopausal symptoms such as vaginal dryness and hot flushes, suggesting a potential direct relationship between menopausal symptoms and psychological problems of cancer patients.

The symptom cluster nausea‐lack of appetite‐diarrhoea (the Gastrointestinal Cluster) was reported in seven studies examining the symptom clusters among patients who had completed treatment.^17^, ^19^, ^22^, ^32^, ^36^, ^38^, ^41^ However, this symptom cluster was less consistently reported among these seven studies, as at least one of the symptoms in the cluster was found to not be associated with this cluster in six of these studies. All seven of these studies showed that additional symptoms can be associated with this cluster, notably certain gastrointestinal symptoms, such as constipation, vomiting and abdominal pain.

Another symptom cluster, comprising hot flushes, vaginal dryness and night sweats (the Menopausal Cluster), was reported in four studies to occur among breast cancer patients who had completed treatment.^17^, ^28^, ^34^, ^36^ Most of these studies showed that the symptoms in this cluster did not cluster independently from other symptoms, except Li et al.^36^ and Marshall et al.,^17^ who identified symptom clusters based on symptoms reported to be of moderate or high severity. Nevertheless, Li et al. showed that only hot flushes and night sweats formed a symptom cluster, while vaginal dryness was not associated with this cluster.^36^

**FIGURE 2:**
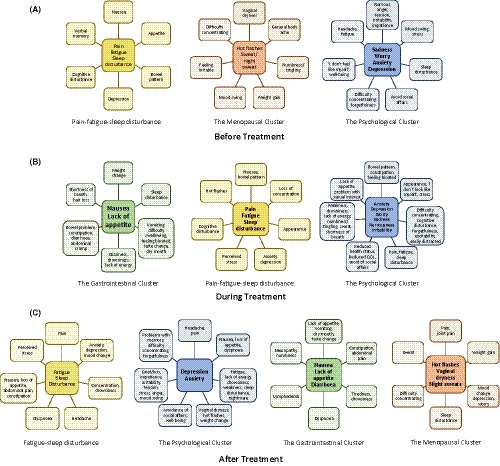
A schematic diagram depicting the symptoms associated with the identified symptom clusters among breast cancer patients before receiving cancer treatment (A), during cancer treatment (B) and after the completion of their cancer treatment (C)

In summary, a number of symptom clusters were identified among breast cancer patients before, during and after cancer treatment. Despite the heterogeneity in the nomenclature of these symptom clusters, four of them, namely Fatigue‐Sleep disturbance, the Psychological Cluster, the Gastrointestinal Cluster and the Menopausal Cluster, were commonly identified by multiple studies. Fatigue‐Sleep disturbance and the Psychological Cluster (anxiety, depression, worry, nervousness, irritability and sadness) were the most common symptom clusters reported by breast cancer patients throughout the course of their disease trajectories, regardless of their stages of treatment. Notably, multiple studies reported that fatigue and/or sleep disturbance and psychological symptoms co‐occurred in patients at all of these treatment stages. In several of the included studies on symptom clusters, patients reported gastrointestinal symptoms including nausea and lack of appetite. These symptoms formed a common cluster (the Gastrointestinal Cluster) that appeared both during and after treatment. Finally, menopausal symptoms, with hot flushes, vaginal dryness and night sweats, formed a cluster (the Menopausal Cluster). According to several studies, some breast cancer patients reported this cluster of symptoms both before and after treatment. In addition to these common symptom clusters, several other clusters were identified in various studies, as summarised in Tables 4, 5 and 6. Figure 2 gives a schematic representation of the symptom clusters identified at various stages of cancer treatment, together with the particular symptoms associated with these clusters.

### 3.4 The longitudinal changes of the composition of symptom clusters

Table 7 shows the longitudinal changes of the composition of the identified symptom clusters as they appeared, at either different stages of cancer treatment or at different phases of the cancer trajectory. In total, 11 of the included studies (34%) involved an assessment of the changes of the composition of symptom clusters over time. Most demonstrated a low level of stability in some of the identified symptom clusters over the course of these studies, with variation in the number of symptoms in the identified clusters at different stages of cancer treatment, resulting in changes in the composition of the symptom clusters over time. Interestingly, Albusoul et al.^37^ even reported that the Gastrointestinal Cluster, comprising the core symptoms of nausea and lack of appetite, disappeared after patients received the fourth cycle of chemotherapy. In addition, symptoms that were initially associated with the Gastrointestinal Cluster before patients underwent treatment, such as pain, fatigue and altered bowel pattern, were found to form a cluster with symptoms in the ‘Treatment‐related’ Cluster after patients commenced treatment. However, the symptoms in this ‘Treatment‐related Cluster’ could then be further divided into two individual clusters after patients had completed treatment. Such division of symptom clusters over time was also observed by Mazor et al.,^34^ who identified symptom clusters based on symptom severity. The changes in the composition of cancer‐associated symptom clusters over time reported in these studies therefore suggests that these clusters are inherently dynamic.

**TABLE 7:** An overview of the included longitudinal studies that identified symptom clusters over the course of the cancer trajectory

Author/year	Identified symptom clusters	Composition of identified symptom clusters at T1	Composition of identified symptom clusters at T2	Composition of identified symptom clusters at T3	Composition of identified symptom clusters at T4
Albusoul et al. (2017)		*Before the start of chemotherapy*	*At the 3rd cycle of chemotherapy*	*At the 4th cycle of chemotherapy*	*After completion of chemotherapy*
	**Gastrointestinal Cluster**	***Nausea***, appetite, bowel pattern, pain, fatigue	***Nausea***, appetite	***Nausea***, bowel pattern, sleep disturbance, pain	
	**Treatment‐related Cluster**	***Sleep disturbance***, ***concentration***, ***anxiety***, ***appearance***	***Sleep disturbance***, pain, ***fatigue***, bowel pattern, ***concentration***, ***appearance***, ***anxiety***, depression	***Fatigue***, appetite, ***concentration***, ***appearan***, ***anxiety***, depression	**1^st^ treatment‐related cluster** ***Fatigue***, ***sleep disturbance***, pain **2^nd^ treatment‐related cluster** ***Concentration***, ***appearance***, ***anxiety***
Berger et al. (2020)		*Before the start of chemotherapy (post‐surgery)*	*1 month after last chemotherapy cycle*	*6 months after last chemotherapy cycle*	
	**Treatment‐related Cluster**	***Sleep disturbance, concentration***, anxiety	Pain, fatigue, ***sleep disturbance, concentration***	Pain, fatigue, ***sleep disturbance, concentration***, anxiety	
	**Gastrointestinal Cluster**	Pain, fatigue, nausea, bowel pattern	Concentration, appearance, anxiety	Pain, bowel pattern	
Browall et al. (2017)		*Before the start of chemotherapy*	*After the 1st cycle of chemotherapy*	*After the 3rd cycle of chemotherapy*	*After completion of chemotherapy*
	**Emotion Cluster**	***Worry***, difficulty concentrating, ***sadness***	***Worry***, difficulty concentrating, ***sadness***	***Worry***, ***sadness***	***Worry***, ***sadness***, nervousness
	**Gastro Cluster**	Taste change, constipation, diarrhoea	Lack of appetite, taste change, constipation, diarrhoea	Mouth sore, dry mouth	Lack of appetite, taste change, constipation, diarrhoea
	**Physical Cluster**	Breathlessness, dizziness, dry mouth, nausea	Breathlessness, dizziness, dry mouth, nausea, hair loss	Lack of appetite, breathlessness, dizziness, nervousness, lack of energy, feeling irritable	Problems with sexual relations, sweats, difficulty sleeping
Chow et al. (2019)		*Before the start of radiotherapy* **Principal component analysis** ***Pain*, *tiredness***, nausea, drowsiness, loss of appetite, dyspnoea Depression, anxiety, well‐being **Exploratory factor analysis** ***Tiredness***, ***drowsiness***, pain, ***nausea***, ***loss of appetite***, ***dyspnoea*** ***Well‐being, depression, anxiety*** **Hierarchical cluster analysis** ***Pain***, tiredness, drowsiness, dyspnoea Depression, anxiety, well‐being ***Nausea, loss of appetite***	*At the end of radiotherapy* **Principal component analysis** ***Pain*, *tiredness***, depression, anxiety and well‐being Nausea, drowsiness, loss of appetite, dyspnoea **Exploratory factor analysis** ***Tiredness***, ***drowsiness*, *nausea***, ***loss of appetite***, ***dyspnoea*** Pain, ***well*‐*being***, ***depression*, *anxiety*** **Hierarchical cluster analysis** ***Pain***, depression, anxiety, well‐being Tiredness, drowsiness, dyspnoea ***Nausea, loss of appetite***	*After radiotherapy* **Principal component analysis** ***Pain*, *tiredness***, depression, anxiety, well‐being, drowsiness, dyspnoea Nausea, loss of appetite **Exploratory factor analysis** Pain, ***tiredness***, ***depression*, *anxiety, well‐being, drowsiness, dyspnoea, nausea, loss of appetite*** **Hierarchical cluster analysis** ***Pain***, tiredness, drowsiness, well‐being, dyspnoea Depression, anxiety ***Nausea, loss of appetite***	
Kenne Sarenmalm et al. (2014)		*At baseline* ***Worry***, ***sadness***, ***nervous***, difficulty sleeping, ***reduced QOL*** and reduced health status Drowsiness, dry mouth, lack of appetite, irritable, difficulty swallowing, shortness of breath Weight loss, ***change in the way food tastes***, constipation, vomiting, hair loss, nausea	*At 1‐month follow‐up* ***Worry***, ***sadness***, ***nervous***, feeling irritable, difficulty concentrating, ‘I don't look like myself’, lack of energy, difficulty sleeping, ***reduced QOL*** Changes in skin, swelling in arms or legs, bloated, numbness/tingling in hands/feet, itching Weight loss, lack of appetite**, *change in which food tastes***, nausea, vomiting, constipation	*At 3‐month follow‐up* ***Worry***, ***sadness***, ***nervous***, feeling irritable, difficulty concentrating, ‘I don't look like myself’, lack of energy, ***reduced QOL***, reduced health status Changes in skin, itching, pain, difficulty swallowing Weight loss, lack of appetite, ***change in which food tastes***, nausea, hair loss	*At 6‐month follow‐up* ***Worry***, ***sadness***, ***nervous***, feeling irritable, difficulty sleeping, feeling bloated**, *reduced QOL***, sweats, pain, problem with sexual interest Changes in skin, vomiting, mouth sores, difficulty swallowing, swelling in arms/legs ***Change in which food tastes***, lack of appetite, lack of energy, drowsiness, dry mouth, hair loss, difficulty concentrating
Kim et al 2008		*Before the start of chemotherapy or radiotherapy*	*After 2nd cycle of chemotherapy or at the final week of the radiotherapy course*	*After 3^rd^ cycle of chemotherapy or 1 month after radiotherapy completion*	
	**Psychoneurological Cluster**	***Depressed mood, cognitive disturbance, fatigue, insomnia, pain***	***Depressed mood, cognitive disturbance, fatigue, insomnia, pain***, hot flashes	***Depressed mood, cognitive disturbance, fatigue, insomnia, pai***	
	**Upper Gastrointestinal Cluster**		***Nausea, vomiting, decreased appetite***	***Nausea, vomiting, decreased appetite***	
Li et al 2020		*Before adjuvant therapy*	*6 months after start of adjuvant therapy*	*12 months after start of adjuvant therapy*	*18 months after start of adjuvant therapy*
	**Psychological Cluster**	***Depression***, anxiety, changes in sleep patterns, avoid of social affairs, ***fatigue***	Anxiety, ***depression, fatigue***, avoid of social affairs	***Fatigue, depression***, changes in sleep patterns	
	**Neurocognitive/Psychonuerocognitive Cluste**r	***Difficulty concentrating, easily distracted, forgetfulness, perceived cognitive disturbance***	***Difficulty concentrating, forgetfulness, easily distracted, perceived cognitive disturbance***, dry mouth	***Easily distracted, difficulty concentrating, perceived cognitive disturbance, forgetfulness***, excitability, tendency toward accidents, short temper, anxiety	***Perceived cognitive disturbance***, excitability, ***forgetfulness***, anxiety,***difficulty concentrating, easily distracted,*** depression, fatigue
	**Musculoskeletal Cluster**	***Joint pain, general aches and pain, muscle stiffnes***	***Joint pain, general aches and pain, muscle stiffness***	***Joint pain, general aches and pain, muscle stiffness***	***Joint pain, general aches and pain, muscle stiffness***
	**Vasomotor Cluster**	***Night sweats, hot flashes***	***Night sweats, hot flashes***	***Night sweats, hot flashes***	***Night sweats, hot flashes***
	**Urinary Cluster**	***Difficulty with bladder control when laughing or crying, difficulty with bladder control at other times***	***Difficulty with bladder control when laughing or crying, difficulty with bladder control at other times***	***Difficulty with bladder control when laughing or crying, difficulty with bladder control at other times***	***Difficulty with bladder control when laughing or crying, difficulty with bladder control at other times***
	**Sexual Cluster**	***Vaginal dryness, pain with intercourse***	***Vaginal dryness, pain with intercourse***	***Vaginal dryness, pain with intercourse***	***Vaginal dryness, pain with intercourse***
	**Weight Cluster**	Decreased appetite, weight loss	Unhappy with the appearance of my body, weight gain	Unhappy with the appearance of my body, weight gain	Unhappy with the appearance of my body, weight gain
	**Gastrointestinal Cluster**		Diarrhoea, nausea		
Mazor et al. (2018)		*Before the start of surgery* **Based on symptom occurrence** ***Anger***, ***impatience***, ***irritability***, ***mood swings, tension*** ***Backache*/*neckache*, *general body aches*, *joint pain or stiffness***, numbness or tingling, painful/tender breast, weight gain Tension, anxiety, depression, difficulty concentrating, ***difficulty falling asleep***, *fatigue*, ***wake during the night*, *waking too early*** ***Hot flashes*, *night sweats***, vaginal dryness**, *daytime sweats*** **Based on symptom severity** ***Anger*, *anxiety*, *depression***, ***difficulty concentrating***, difficulty falling asleep**, *fatigue*, *forgetfulness***, headache, ***impatience***, ***irritability***, ***mood swings*, *tension***, waking during the night, waking too early General body aches, ***daytime sweats*, *night sweats*, *hot flashes***, vaginal dryness, numbness/tingling, weight gain ***General body aches***, numbness/tingling, ***backache*/*neckache*, *joint pain and stiffness***	*12 months after surgery* **Based on symptom occurrence** ***Anger***, anxiety, depression, difficulty concentrating, fatigue, forgetfulness, ***impatience***, ***irritability***, ***mood swings*, *tension*** ***Backache/neckache, general body aches, joint pain or stiffness*** ***Difficulty falling asleep, wake during the night, waking too early*** ***Hot flashes, night sweats, daytime sweats*** **Based on symptom severity** ***Anger, anxiety, depression***, ***impatience***, ***irritability***, ***mood swings, tension*** ***Difficulty concentrating*, *fatigue*, *forgetfulness***, painful/tender breasts ***Daytime sweats, night sweats, hot flashes*** ***General body aches***, headache, ***backache*/*neckache*, *joint pain and stiffness*** Difficulty falling asleep, wake during the night, wake too early		
Phligbua et al. (2013)		*Before the start of chemotherapy*	*After the 1st cycle of chemotherapy*	*After completion of chemotherapy*	
	**Menopausal Cluster**	***Sweats, night sweats, hot flashes,*** ***mood swings***, feeling irritable, ***difficulty concentrating***		Difficulty sleeping, ***sweat, hot flashes, night sweats, difficulty concentrating***, pain, worry	
	**Discomfort Symptom Cluster**	Dizziness, joint pain, vaginal itching/irritation, constipation	Constipation, urinary problem, difficulty sleeping, feeling bloated	Numbness/tingling in hands/feet, dry mouth	
	**Post‐operative Symptom Cluster**	Cough, itchiness, numbness/tingling in hands/feet			
	**Fatigue Cluster**	Difficulty sleeping, lack of energy			
	**Gastrointestinal‐related Fatigue Cluster**		***Lack of energy***, nausea, ***lack of appetite, drowsiness***, dizziness, ***taste changes***	***Lack of energy, drowsiness, lack of appetite, taste change***	
	**Psychological Cluster**	Sadness, worry			
	**Disturbed in Mood Symptom Cluster**		***Feeling irritable***, pain, nervousness	Mood swings, *****feeling irritable*****, joint pain	
	**Psychologically‐related Self‐image Cluster**		Skin changes, ‘I don't look like myself’, worry, difficulty concentrating, hair loss		
	**Self‐image Symptom Cluster**			Skin changes, hair loss, ‘I don't look like myself’	
	**Oral Cluster**		Mouth sore, dry mouth		
Starkweather et al. (2017)		*Before the start of adjuvant chemotherapy*	*Before 4th cycle of adjuvant chemotherapy*	*After completion of adjuvant chemotherapy*	
	**Global Cognition Cluster**	***Cognitive flexibility***, ***executive functioning***, ***complex attention***, ***reaction time***, processing speed	***Cognitive flexibility***, ***executive functioning***, ***complex attention***, ***reaction time***, processing speed, psychomotor speed, pain	***Cognitive flexibility***, ***executive functioning***, ***complex attention***, ***reaction time***	
	**Affective Symptom Cluster**	***Perceived stress***, ***anxiety***, ***depression***, ***sleep disturbance***, ***fatigue***	***Perceived stress***, ***anxiety***, ***depression***, ***sleep disturbance***, ***fatigue***	***Perceived stress***, ***anxiety***, ***depression***, ***sleep disturbance***, pain, ***fatigue***	
	**Cognitive Efficiency Cluster**	Sleep disturbance, fatigue, ***pain***, verbal memory	Processing speed, psychomotor speed, ***pain***, verbal memory	Processing speed, reaction time, psychomotor speed, ***pain***, fatigue	
	**An additional cluster was also identified:**	***Psychomotor speed***, ***visual memory***, processing speed	***Psychomotor speed***, ***visual memory***	***Psychomotor speed***, ***visual memory***, verbal memory	
Ward Sullivan et al. (2018)		*Before the start of chemotherapy* **Based on symptom occurrence**	*1 week after start of chemotherapy* **Based on symptom occurrence**	*2 weeks after start of chemotherapy* **Based on symptom occurrence**	
	**Sickness Behavior Symptom Cluster**	Pain, dry mouth, nausea, drowsiness, numbness/tingling, lack of appetite, dizziness			
	**Psychological Symptom Cluster**	Difficulty concentrating, ***nervousness, sadness, worry, irritability***, ‘I don't look like myself’	***Nervousness, sadness, worry, irritability***, ‘I don't look like myself’	***Nervousness, sadness, worry, irritability***, difficulty concentrating, drowsiness	
	**Hormonal Symptom Cluster**	***Hot flashes, sweats***	***Hot flashes***, difficulty sleeping, ***sweats***, problem with sexual interest or activity	***Hot flashes, sweats***	
	**Gastrointestinal Symptom Cluster**	Difficulty sleeping, abdominal cramps, shortness of breath, weight loss	Weight loss, feeling bloated, weight gain	Abdominal cramps, difficulty sleeping, feeling bloated, weight gain, nausea	
	**Weight Change Symptom Cluster**	Weight loss, weight gain			
	**Epithelial Symptom Cluster**	Weight gain, ***mouth sores,*** hair loss, ***change in the way food tastes***, change in skin	“I don't look like myself”, ***change in the way food tastes***, hair loss, ***mouth sores***	***Change in the way food tastes***, changes in skin, itching, ***mouth sores***, “I don't look like myself”	
	**Nutritional Symptom Cluster**		Dry mouth, ***nausea, lack of appetite, change in the way food tastes, weight loss***, abdominal cramps, diarrhoe	Weight gain, ***nausea, lack of appetite, weight loss, change in the way food tastes***	
			
		**Based on symptom severity**	**Based on symptom severity**	**Based on symptom severity**	
	**Sickness Behavior Symptom Cluster**	Pain, dry mouth, nausea, drowsiness, dizziness			
	**Psychological Symptom Cluster**	Difficulty concentrating, ***nervousness, sadness, worry, irritability***, 'I don't look like myself'	***Nervousness, sadness, worry, irritability***	Difficulty concentrating, ***nervousness, sadness***, drowsiness, ***worry, irritability***	
	**Hormonal Symptom Cluster**	***Sweats, hot flashes***	***Sweats, hot flashes***	***Hot flashes, sweats***	
	**Gastrointestinal Symptom Cluster**	***Feeling bloated***, diarrhoea, ***abdominal cramps***	***Feeling bloated, abdominal cramps***, weight gain	***Feeling bloated, abdominal cramps***, weight gain	
	**Weight Change Symptom Cluster**	lack of appetite, weight gain, weight loss			
	**Epithelial Symptom Cluster**	***'I don't look like myself'***, weight gain, ***hair loss, change in the way food tastes, changes in skin***	***Hair loss, change in the way food tastes, 'I don't look like myself', changes in skin***, mouth sores	***Change in the way food tastes***, mouth sores, ***hair loss, 'I don't look like myself', changes in skin***	
	**Chemotherapy‐Neuropathy Symptom Cluster**		Drowsiness, numbness in hands/feet, pain		
	**Nutritional Symptom Cluster**		***Weight gain, weight loss, nausea, lack of appetite***	***Weight gain, nausea, lack of appetite, weight loss***, change in the way food tastes	

Symptoms shown in bold and italics are those that appear in the same symptom cluster at all time points of symptom assessment.

Although most of the studies showed that the composition of symptom clusters was generally unstable over time, some clusters identified in these studies exhibited a degree of stability in their composition. For example, Kim et al.^39^ demonstrated that the symptoms of depressed mood, cognitive disturbance, fatigue, insomnia and pain, which form the ‘Psychoneurological’ Cluster, remained associated and clustered with each other both before and during cancer treatment. The composition of the ‘Upper Gastrointestinal’ Cluster, comprising nausea, vomiting and decreased appetite, also remained unchanged at two different time points during cancer treatment. Further, both Browall et al.^38^ and Kenne Sarenmalm et al.^27^ showed that the composition of the Psychological Cluster remained generally stable over time, with core symptoms such as sadness and worry appearing in the cluster at every time point of symptom assessment. Likewise, the ‘Global Cognition’ Cluster and ‘Affective’ Cluster identified by Starkweather et al.^30^ appeared generally stable, with the majority of the core symptoms remaining unchanged before, during and after cancer treatment. Moreover, certain uncommon symptom clusters identified by Li et al.,^33^ including the ‘Neurocognitive‐Psychoneurocognitive’ Cluster, ‘Musculoskeletal’ Cluster, ‘Vasomotor’ Cluster, ‘Sexual’ Cluster and ‘Urinary’ Cluster, remained generally stable among patients over the 18 months of cancer treatment. Interestingly, however, Chow et al.^32^ showed that whereas symptom clustering through exploratory factor analysis yielded generally stable symptom clusters among patients pre‐ and post‐radiotherapy treatment, symptom cluster identification via principal component analysis or hierarchical cluster analysis did not. Such a finding lends further support to the observation that the methodologies used for cluster analysis can lead to variations in cluster identification in symptom cluster studies.

Overall, this review of the 32 included studies demonstrated that most of the cancer‐associated symptom clusters exhibited a low level of compositional stability over time, with individual symptoms forming different clusters at different stages of cancer treatment.

## 4 DISCUSSION

### 4.1 Symptom clusters among breast cancer patients

Our review provides an overview of a number of common symptom clusters that were identified in studies of breast cancer patients. This overview shows that Pain‐Fatigue‐Sleep disturbance, the Psychological Cluster, the Gastrointestinal Cluster and the Menopausal Cluster are among the most common symptom clusters identified. One notable finding is that the Fatigue‐Sleep disturbance and Psychological Clusters were often reported among patients at all three stages across the cancer treatment process, and even before the start of cancer treatment. These findings indicate that these symptom clusters are likely to result from both the cancer itself and from the detrimental effects of its treatment. Specifically, pain, fatigue and sleep disturbance were commonly found to co‐occur, both before and during cancer treatment. This observation is consistent with previous findings, and it suggests that these symptoms are among the most prevalent in cancer patients receiving treatment.^45^ Interestingly, a number of the included studies reported that Fatigue‐Sleep disturbance continued to affect cancer patients even after they had completed treatment. Two of the studies showed that pain was associated with this cluster (Table 6). Moreover, studies involving longitudinal assessments of the symptoms experienced by breast cancer patients revealed the persistence of the clustering of pain, sleep disturbance and fatigue symptoms, both during cancer treatment and after its completion.^37^, ^39^ Likewise, the studies involving longitudinal assessments of symptoms found that psychological symptoms, in particular anxiety and depression, were present before, during and/or after treatment.^30^, ^32^, ^33^, ^34^ More importantly, these two psychological symptoms were previously suggested to co‐occur with Pain‐Fatigue‐Sleep disturbance, and the severity of each symptom cluster was exacerbated by the occurrence of another.^46^ All of these observations suggest the importance of developing effective interventions to target both Pain‐Fatigue‐Sleep disturbance and psychological symptoms. Furthermore, these findings underscore the need for persisting with such interventions even after patients complete treatment, as a means to safeguard their ongoing well‐being.

Another question raised in this review is why certain symptom clusters, such as Pain‐Fatigue‐Sleep disturbance and the Psychological Cluster, tend to co‐occur across the cancer trajectory. Such co‐occurrence of symptom clusters could potentially be caused by alterations in certain molecular pathways associated with these two clusters, such as the dysregulation of HPA axis functioning, altered serotonin neurotransmission or increased pro‐inflammatory cytokine production.^47^, ^48^, ^49^, ^50^, ^51^, ^52^, ^53^, ^54^, ^55^ Indeed, a previous review had also demonstrated that pro‐inflammatory cytokines and immune markers could be related to the clustering of symptoms associated with cancer treatment.^56^ It is likely that symptoms in these clusters are caused by common biological pathways mentioned above, so that alterations in these pathways may lead to concurrent expression of both symptom clusters. This pattern of shared pathways could potentially explain why these two symptom clusters often co‐occur. Nevertheless, additional research is required to confirm this hypothesis, and to dissect further molecular pathways linked to the development of these symptom clusters.

Many studies have demonstrated the detrimental effect of symptom clusters on the QOL and/or functional status of breast cancer patients.^6^, ^57^, ^58^, ^59^ With Pain‐Fatigue‐Sleep disturbance and the Psychological Cluster shown to be some of the most common symptom clusters among breast cancer patients, tailored interventions capable of targeting both clusters need to be developed for QOL improvement of breast cancer patients. Over the past few years, numerous studies have examined the effectiveness of certain non‐pharmacological interventions in managing such symptom clusters. These interventions include mindfulness‐based stress reduction,^60^ cognitive behavioural therapy,^45^ guided imagery intervention^61^ and certain Chinese medical practices such as acupuncture^62^ and Tai Chi Qigong.^63^ The effectiveness of these interventions for managing symptom clusters was demonstrated by these studies.^64^ Furthermore, a systematic review has suggested that psychoeducational interventions, which involve information sharing, training on problem‐solving and coping skills and psychosocial support, may alleviate symptom clusters and significantly improve QOL.^65^ Given the demonstrated effectiveness of the above‐described interventions, healthcare providers should consider using interventions involving a mixture of these components as an integral part of post‐treatment care for cancer patients.

One major observation of this review is the high level of heterogeneity in the types of symptom clusters identified in the included studies. Even when studies report the same symptom clusters, the composition of these clusters varies considerably. There are two possible reasons for such variations. First, study participants underwent different cancer treatment regimens. As indicated in Table 3, a substantial number of the studies comprised a mixture of treatment types, such as chemotherapy, radiotherapy or hormonal therapy, rather than a specific type of treatment. Variations in treatment type could have resulted in different symptom experiences,^58^, ^66^, ^67^ and possibly the co‐occurrence of different symptoms among these participants, resulting in variations in the composition of the reported symptom clusters.

Second, the methodology used for symptom‐cluster identification appeared to vary between the studies. As indicated above, different sets of symptoms were found to cluster together if symptom clustering was based on different parameters of symptom experience, such as symptom occurrence, severity and distress. Moreover, the use of different instruments for assessing the participants’ symptom experience for symptom cluster identification could also have a similar effect on clustering. Notably, a wide range of instruments was used in studies (Table 3). For example, Matthews et al.^14^ and Phligbua et al.^28^ reported differences in the composition of the Gastrointestinal Cluster (nausea‐lack of appetite), demonstrating variations in the additional symptoms that were associated with this cluster. Such variations may be attributable to the fact that while Matthews et al. utilised the Symptom Distress Scale, Phligbua et al. used the modified Memorial Symptom Assessment Scale for symptom cluster identification. As indicated by Kim et al.,^68^ different symptom assessment instruments each assess a specific range of symptoms. As a result, the use of different instruments may have contributed to the different sets of symptoms that were found to be associated with a given cluster.

### 4.2 Instability of composition of symptom clusters over time

Another notable finding of this review is that the composition of symptom clusters among breast cancer patients appears to change over time. A considerable number of the symptom clusters identified in the included longitudinal studies showed changes in the numbers and types of symptoms, both prior to treatment and at various stages of cancer treatment. Such variability did not always appear, as Kim et al.^39^ found that the composition of the identified symptom clusters remained generally unchanged, and Li et al.,^33^ Starkweather et al.,^30^ Mazor et al.,^34^ and Ward Sullivan et al.^35^ found a fair level of stability in some of the identified symptom clusters. Overall, the small number of symptoms in these stable symptom clusters might explain their apparent stability. Our findings on the temporal instability of symptom clusters were consistent with those of a previous review on symptom clusters among advanced cancer patients.^3^ Furthermore, these findings generally agreed with those reported in a review by Ward Sullivan et al..^69^ In that review, 60% of the included longitudinal studies observed instability of the identified symptom clusters among cancer patients receiving chemotherapy. Although the causes of the dynamic nature of symptom clusters are still not fully understood, Kirkova and Walsh^7^ previously proposed that changes in symptom severity over time could potentially offer an explanation. In support of this hypothesis, a recent study demonstrated in a cohort of gastrointestinal cancer patients that the severity of symptoms may change at different stages of cancer treatment.^5^ Indeed, perceived symptom severity is one of the most widely‐used symptom experience parameters used for assessment during *de novo* identification of symptom clusters.^70^ As the severity of the assessed symptoms changes over time, it is possible that the extent to which certain symptoms show an association with a cluster can vary at different time points of symptom assessment. This would result in different symptoms clustering to form a given cluster at various stages of treatment, as demonstrated in this review.

Physiological changes in patients during treatment offer another potential explanation for the dynamic nature of symptom clusters. As indicated above, symptom clusters can result from the deregulation of certain molecular pathways, such as inflammation caused by the increased production of pro‐inflammatory cytokines. Indeed, pain, fatigue, sleep disturbance and depression, previously identified as symptoms of the Psychoneurological Cluster, were shown to be associated with these pro‐inflammatory events, and the severity of these symptoms may be modulated by the production level of these pro‐inflammatory mediators. It is possible that the extent of these events, as indicated by the level of pro‐inflammatory cytokine production, may be modulated throughout the course of cancer treatment, in turn modulating the severity of the aforementioned symptoms. Given the possible effect of symptom severity in the formation of symptom clusters, as explained above, it is likely that such physiological changes may also contribute to the changes in symptom cluster composition during the treatment regimens. Nevertheless, this hypothesis needs to be confirmed by further studies.

In light of the possibility of changes in symptom cluster composition over time, further research efforts should examine the longitudinal changes of clusters, preferably with symptoms assessed at every treatment stage. This line of research would enable the optimal tailoring of symptom management interventions for cancer patients at various stages of treatment, which would facilitate the development of more effective oncology care plans tailored to patients’ individual needs.

### 4.3 Future work

To facilitate the formulation of effective oncology care plans, future work should also be directed towards exploring the molecular mechanisms involved in the occurrence of symptom clusters. A deeper understanding of the mechanistic aspects of symptom clusters would provide invaluable insights into how more effective symptom management interventions may be developed using pharmacological or non‐pharmacological strategies that target the identified biological mechanisms and pathways. Moreover, identification of the symptom cluster‐associated pathways could provide clues for identifying biomarkers that could be targeted to address those symptom clusters. Such an approach could facilitate the development of improved symptom management interventions.

Although studies have provided clues to the aetiology of symptom clusters and revealed potential molecular pathways that may be associated with certain symptom clusters, more studies are required to fully validate these findings and explore other mechanisms that may be associated with the currently known symptom clusters. These studies would reveal any common biological pathways that are associated with various symptom clusters experienced by patients, enabling the development of effective interventions for managing multiple symptom clusters.

### 4.4 Limitations

This review has two major limitations. First, only articles published in English were included in this review, and therefore symptom clusters reported in articles that were published in other languages were not included for analysis in this review. Second, there is a high degree of heterogeneity in the methodology used for symptom assessment of patients and symptom cluster identification between the included studies. As reported by Chow et al.,^32^ different forms of cluster analyses utilised for symptom cluster identification would result in variations in the composition of the identified symptom clusters. Caution is therefore required for the interpretation of the findings of this review.

## 5 CONCLUSIONS

As mounting evidence suggests that cancer‐associated symptoms often co‐occur and that these symptoms can mutually affect their occurrence and severity, more studies have aimed to identify cancer‐associated symptom clusters. Our review provides an overview of the identified symptom clusters among breast cancer patients, and reveals that Fatigue‐Sleep disturbance and the Psychological Cluster (such as anxiety, depression, sadness, worry, nervousness and irritability) are two of the most commonly reported symptom clusters among these individuals. Some of these symptom clusters also exhibit a considerable degree of longitudinal instability, as evidenced by the substantial changes in their composition across the various stages of cancer treatment.

Nevertheless, inconsistencies exist in the findings between the included studies, in terms of the number of additional symptoms that are associated with a particular symptom cluster, primarily owing to the heterogeneity of the methodologies used by studies for symptom cluster identification. Such heterogeneity hampers the drawing of definitive conclusions on which symptom clusters would most likely occur among breast cancer patients at a particular treatment stage. Future studies should therefore examine symptom clusters separately among patients undergoing a particular treatment type, and use standardised instruments for symptom assessment during symptom cluster identification. Moreover, further studies should be conducted to reveal the biological pathways associated with the occurrence of various symptom clusters, by examining the association between the expression level of certain biological markers and the severity of symptom clusters. Such studies would help us explore the common biological pathways underpinning these symptom clusters and provide valuable information on effective strategies for targeting these pathways. Ultimately, this would provide useful clues for the development of effective, patient‐tailored interventions for managing multiple symptoms at a minimal cost.

## CONFLICTS OF INTEREST

The authors report no conflicts of interest in this work.

## AUTHOR CONTRIBUTIONS

Winnie K.W. So, Xiaole He, Dorothy N.S. Chan, Carmen W.H. Chan and Alexandra L. McCarthy set the aim and focus of the review. Bernard M.H. Law and Marques S.N. Ng did the literature search, data extraction and critical appraisal. Bernard M.H. Law drafted the manuscript. Winnie K.W. So, Xiaole He, Marques S.N. Ng, Dorothy N.S. Chan, Carmen W.H. Chan and Alexandra L. McCarthy critically reviewed and revised the manuscript. All authors approved the final version of the manuscript.

## ETHICAL APPROVAL

The manuscript is a systematic review. Ethical approval is not required for the conduction of the systematic review.

## Data Availability

This article is a systematic review. Data sharing is not applicable to this article as no new data were created or analysed in this study.
